# Naringin Attenuates High Fat Diet Induced Non-alcoholic Fatty Liver Disease and Gut Bacterial Dysbiosis in Mice

**DOI:** 10.3389/fmicb.2020.585066

**Published:** 2020-11-13

**Authors:** Hongna Mu, Qi Zhou, Ruiyue Yang, Jie Zeng, Xianghui Li, Ranran Zhang, Weiqing Tang, Hongxia Li, Siming Wang, Tao Shen, Xiuqing Huang, Lin Dou, Jun Dong

**Affiliations:** ^1^The Key Laboratory of Geriatrics, Beijing Institute of Geriatrics, Beijing Hospital, National Center of Gerontology, National Health Commission, Institute of Geriatric Medicine, Chinese Academy of Medical Sciences, Beijing, China; ^2^National Center for Clinical Laboratories, Beijing Hospital, National Center of Gerontology, Institute of Geriatric Medicine, Chinese Academy of Medical Sciences, Beijing, China

**Keywords:** citrus fruits, lipogenesis, gut dysbiosis, high-fat diet, non-alcoholic fatty liver disease

## Abstract

The incidence of non-alcoholic fatty liver disease (NAFLD) is rising annually, and emerging evidence suggests that the gut bacteria plays a causal role in NAFLD. Naringin, a natural flavanone enriched in citrus fruits, is reported to reduce hepatic lipid accumulation, but to date, no investigations have examined whether the benefits of naringin are associated with the gut bacteria. Thus, we investigated whether the antilipidemic effects of naringin are related to modulating the gut bacteria and metabolic functions. In this study, C57BL/6J mice were fed a high-fat diet (HFD) for 8 weeks, then fed an HFD with or without naringin administration for another 8 weeks. Naringin intervention reduced the body weight gain, liver lipid accumulation, and lipogenesis and attenuated plasma biochemical parameters in HFD-fed mice. Gut bacteria analysis showed that naringin altered the community compositional structure of the gut bacteria characterized by increased benefits and fewer harmful bacteria. Additionally, Spearman’s correlation analysis showed that at the genus level, *Allobaculum*, *Alloprevotella*, *Butyricicoccus*, *Lachnospiraceae_NK4A136_group*, *Parasutterella* and *uncultured_bacterium_f_Muribaculaceae* were negatively correlated and *Campylobacter*, *Coriobacteriaceae_UCG-002*, *Faecalibaculum* and *Fusobacterium* were positively correlated with serum lipid levels. These results strongly suggest that naringin may be used as a potential agent to prevent gut dysbiosis and alleviate NAFLD.

## Introduction

Non-alcoholic fatty liver disease (NAFLD) is a common, multifactorial, and poorly understood liver disease whose incidence is rising globally. NAFLD is mainly involved with unhealthy dietary patterns and lifestyles (Aron-Wisnewsky et al., 2020). Accumulating evidence demonstrates that cardiovascular diseases and type II diabetes are closely associated with NAFLD progression, placing an increasing burden on society (Petta et al., 2015). Although often clinically silent, NAFLD can progress to non-alcoholic steatohepatitis, cirrhosis and end-stage liver disease over time (Pais et al., 2016). NAFLD-related liver failure has become the second leading cause of liver transplantation in western countries (Glass et al., 2020). Lifestyle modification is the mainstay of treatment, including dietary changes and exercise, with the primary goal being weight loss. Developing medicines and nutritional foods to prevent NAFLD remains a challenge for all scientists (Zhang et al., 2016).

Non-alcoholic fatty liver disease development and progression depends on pathologic accumulation of lipid droplets within hepatocytes (Yan et al., 2014; Lei et al., 2020). At the molecular level, sterol regulatory element binding protein 1 (Srebp1) is a key lipogenic transcription factor that directly regulates the expressions of lipid synthesis rate-limiting enzymes, including fatty acid synthase (Fas), acetyl-CoA carboxylase (Acc), and stearoyl-CoA desaturase 1 (Scd1), and lipid uptake-related genes, such as CD36, leading to hepatic lipid accumulation (Guo et al., 2018; Zhou et al., 2020). Importantly, expression levels of these genes in the liver are upregulated in NAFLD-model mice, suggesting a crucial role of lipid synthesis in hepatic steatosis (Fu et al., 2018; Zhang et al., 2020).

Scholars have recently begun studying the gut bacteria to understand NAFLD development and progression. The gut bacteria affects lipid metabolism and lipid levels in blood and tissues, both in mice and humans (Yin et al., 2018; Schoeler and Caesar, 2019). Transplanting the gut bacteria isolated from high-fat diet (HFD)-induced obese donors to germ-free animals led to increases in body weight and metabolic syndrome in the recipient mice (Turnbaugh et al., 2008). In addition, germ-free mice devoid of a gut bacteria are resistant to diet-induced obesity, steatosis, and insulin resistance (Le Roy et al., 2013). Therefore, determining the gut bacteria composition may help predict NAFLD severity and suggest novel therapeutic targets (Boursier et al., 2016).

Naringin (NAR), a principal flavanone enriched in citrus fruits, appears to reduce hepatic lipid accumulation (Raffoul-Orozco et al., 2018; Zhou et al., 2019), giving it great therapeutic potential for treating NAFLD. NAR is reported to exhibit antihyperglycemic and antioxidant properties (Ahmed et al., 2017; Rotimi et al., 2018). However, the mechanisms by which NAR acts on lipid accumulation remain unclear. Daily consumption of orange (which contains hesperidin and NAR) may positively affect the gut microbial and metabolic biomarkers in young women (Lima et al., 2019). Thus, using NAR to treat NAFLD might be a potential strategy to modulate the gut bacteria composition. However, no studies have reported evaluating the effect of NAR on the gut bacteria in NAFLD-model mice. This study assessed the effectiveness of treatment with NAR on the gut bacteria in NAFLD-model mice for the first time.

## Materials and Methods

### Animals

Six-week-old male C57BL/6J mice were purchased from Vital River Laboratory Animal Technology Co., Ltd. (Beijing, China) and housed in polypropylene cages (*n* = 4 mice/cage). Animals were housed at 20 ± 2°C on a 12-h light/dark cycle and allowed free access to food and water. Throughout the experiment, the bedding and water were changed once weekly, and the HFD was changed twice weekly to prevent fat oxidation, which produced an odor that affected the eating habits of the mice.

### Experimental Protocols and Groups

The normal chow diet (ND, 1025), the ND supplemented with 0.07% NAR (Sigma-Aldrich, St. Louis, MO, United States), the HFD (synthetic diet supplemented with 0.15% cholesterol, w/w and 41% energy from fat, H10141), and the HFD supplemented with 0.07% NAR were purchased from Beijing HFK Bioscience Co., Ltd. (Beijing, China). The dose of 0.07% NAR is based on previous studies (Wang et al., 2015; Sui and Xiao, 2018). After 1 week of acclimation on ND, the mice were randomly divided into the ND group (*n* = 20) and the HFD group (*n* = 20) and treated for 8 weeks. Mice in the ND group were then randomly divided into the ND and ND+NAR groups (*n* = 10 mice per group), and the mice in the HFD group were randomly divided into the HFD and HFD+NAR groups (*n* = 10 mice per group). The ND group was fed ND, and the ND+NAR group was fed ND containing 0.07% NAR. The HFD group was fed a normal HFD, and the HFD+NAR group was fed an HFD supplemented with 0.07% NAR. Body weight was measured every 2 weeks. After 8 weeks, the mice were sacrificed after collecting blood samples and liver tissues. Ileal, cecal, and colonic contents were aseptically collected and immediately stored at −80°C until use. The Peking University Biomedical Ethics Committee Experimental Animal Ethics Branch (LA2013-73) approved all protocols for the diets, the anesthesia, the blood and tissue sample collection, and disposal of the dead animals. The protocols conformed to the Guide for the Care and Use of Laboratory Animals (National Institutes of Health).

### Biochemical Assays

The blood samples were centrifuged at 3500 rpm for 10 min at 4°C to separate the serum. The serum total cholesterol (TC), triglycerides (TG), low-density lipoprotein cholesterol (LDL-C), high-density lipoprotein cholesterol (HDL-C), alanine aminotransferase (ALT), aspartate aminotransferase (AST), glucose and high-sensitivity C-reactive protein (hsCRP) contents were measured with a 7180 automatic biochemical analyzer (Hitachi Ltd., Tokyo, Japan).

### Enzyme-Linked Immunosorbent Assay

Enzyme-linked immunosorbent assay (ELISA) was conducted using specific kit to determine the levels of lipopolysaccharides (LPS, Cloud-Clone Corp, Houston, TX, United States) in serum.

### Histological and Immunohistochemistry Analysis

Liver tissue samples were fixed in 4% paraformaldehyde, routinely processed, embedded in paraffin, sliced in 5-μm sections, and stained with hematoxylin and eosin (HE) for histological analysis. Frozen sections (8-μm) were stained with oil red O. Immunohistochemistry staining of myeloperoxidase (MPO, Thermo Scientific, Fremont, CA, United States) and F4/80 (Cell Signaling Technology, Beverly, MA, United States) were performed using standard procedures.

### Western Blotting Assay

Total protein was extracted using a protein extraction kit (Applygen Technologies, Beijing, China). The protein concentration was determined with a BCA protein assay kit (Applygen Technologies, Beijing, China). Western blot analysis was performed routinely, with primary antibodies against β-actin, Acc, Fas (Cell Signaling Technology, Beverly, MA, United States), Scd1 and Srebp1 (Thermo Scientific, Fremont, CA, United States). 100 μg protein was loaded in each well. The bands were detected using an ECL detection kit (Applygen Technologies, Beijing, China). For quantification, band intensity was assessed by densitometry and expressed as the mean area density using Quantity One image analyzer software (Bio-Rad, Richmond, CA, United States).

### PCR Amplification and Sequencing

To amplify the V3–V4 region of the 16S rRNA gene for Illumina deep sequencing, the universal primers, 338F: 5′-ACTCCTACGGGAGGCAGCA-3′ and 806R: 5′-GGACTACHVGGGTWTCTAAT-3′, were used. PCR was performed in a total reaction volume of 20 μL: 13.25 μL H_2_O, 2.0 μL 10 × PCR Ex Taq Buffer, 0.5 μL DNA template (100 ng/mL), 1.0 μL primer 1 (10 mmol/L), 1.0 μL primer 2 (10 mmol/L), 2.0 μL dNTP and 0.25 μL Ex Taq (5 U/mL). After an initial denaturation at 95°C for 5 min, amplification was performed with 30 cycles of incubation for 30 s at 95°C, 20 s at 58°C, and 6 s at 72°C, followed by a final extension at 72°C for 7 min. The amplified products were purified and recovered using 1.0% agarose gel electrophoresis. Beijing Biomarker Technologies Co., Ltd. (Beijing, China) performed the library construction and sequencing.

### Bioinformatics Analysis

Paired-end reads were merged using FLASH v1.2.7 (Magoč and Salzberg, 2011); tags with >6 mismatches were discarded. Merged tags with an average quality score <20 in a 50-bp sliding window were trimmed by Trimmomatic (Bolger et al., 2014), and those shorter than 350 bp were removed. Possible chimeras were further removed, and the denoised sequences were clustered into operational taxonomic units (OTUs) with 97% similarity using USEARCH (version 10.0). Taxonomy was assigned to all OTUs by searching against the Silva databases (Release 128) using QIIME software. The alpha diversity (i.e., ACE, Chao1, and Shannon diversity) and beta diversity (i.e., binary Jaccard distance-based principal coordinate analysis) were analyzed using QIIME.

### Statistical Analysis

All data are expressed as the mean ± SEM. Statistical analysis was performed using one-way analysis of variance followed by Tukey’s *post hoc* test. Correlations between filtered fecal bacteria and serum lipid levels were calculated using the Spearman rank correlation. *P* < 0.05 was considered statistically significant.

## Results

### NAR Supplementation Reduced HFD-Induced Lipid Accumulation in Mice

**FIGURE 1:**
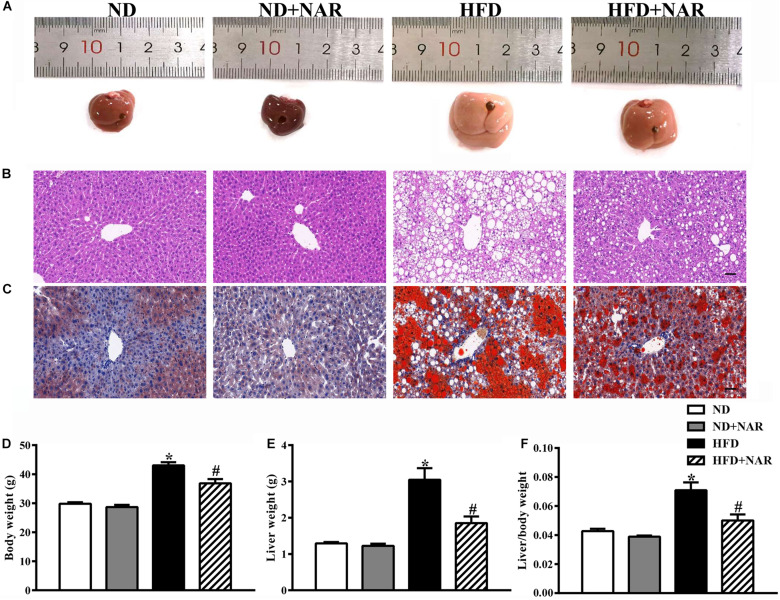
Effects of NAR on fat accumulation and body weight in HFD-fed mice. **(A)** Macroscopic view of livers from the different groups. Representative images of light microscopic **(B)** HE and **(C)** oil red O staining of liver tissues in the different groups, Bar = 50 μm. **(D)** Body weight, **(E)** liver weight, and **(F)** liver/body weight. Data are the mean ± SEM (*n* = 8). *P < 0.05 vs. ND group; #*P* < 0.05 vs. HFD group.

The livers in the HFD group displayed more lipid accumulation with an enlarged volume, yellow color and hard texture compared with the livers of the ND group, which exhibited a soft texture with a smooth, red-brown surface (Figure 1A). Administering NAR after the HFD greatly improved liver manifestations. HE and oil red O staining showed more hepatocytes with ballooning degeneration and more red-stained lipids in the HFD group; however, lipid accumulation in the liver tissue was reduced in the HFD+NAR group (Figures 1B,C). Additionally, HFD-fed mice gained more body weight, liver weight, and liver/body weight than did the ND-fed mice. NAR supplementation prevented this gain in the HFD-fed mice (Figures 1D–F). Thus, NAR ameliorated the HFD-induced liver lipid accumulation and weight gain.

### NAR Supplementation Ameliorated Blood Lipid Levels

**FIGURE 2:**
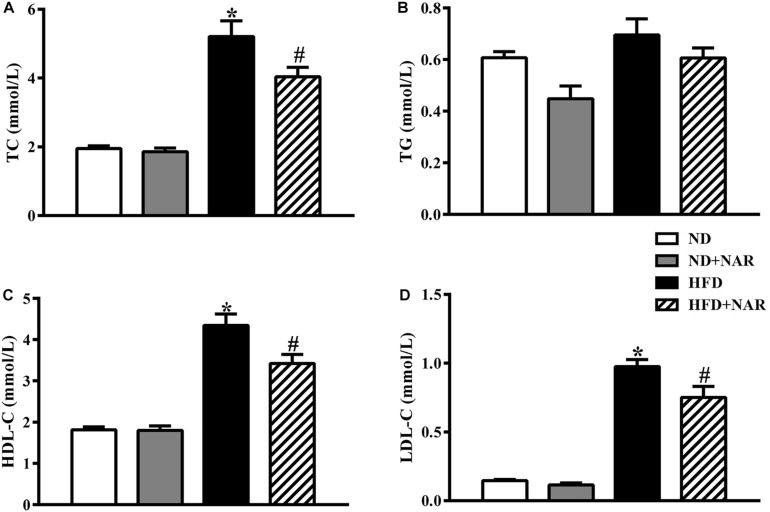
Effects of NAR on serum lipid levels in HFD-fed mice. Serum levels of **(A)** TC, **(B)** TG, **(C)** HDL-C, and **(D)** LDL-C. Data are the mean ± SEM (*n* = 8). *P < 0.05 vs. ND group; #*P* < 0.05 vs. HFD group.

Figure 2 shows the blood lipid results of the mice from all four groups. Compared with the control group, HFD-fed mice showed increased TC, HDL-C, and LDL-C levels, indicating that the HFD group had abnormal blood lipid metabolism. NAR supplementation protected against HFD-induced hyperlipidemia. Serum TG levels did not differ among the four groups.

### NAR Supplementation Attenuated Liver Function, Serum Glucose, hsCRP and LPS and Liver Inflammation

**FIGURE 3:**
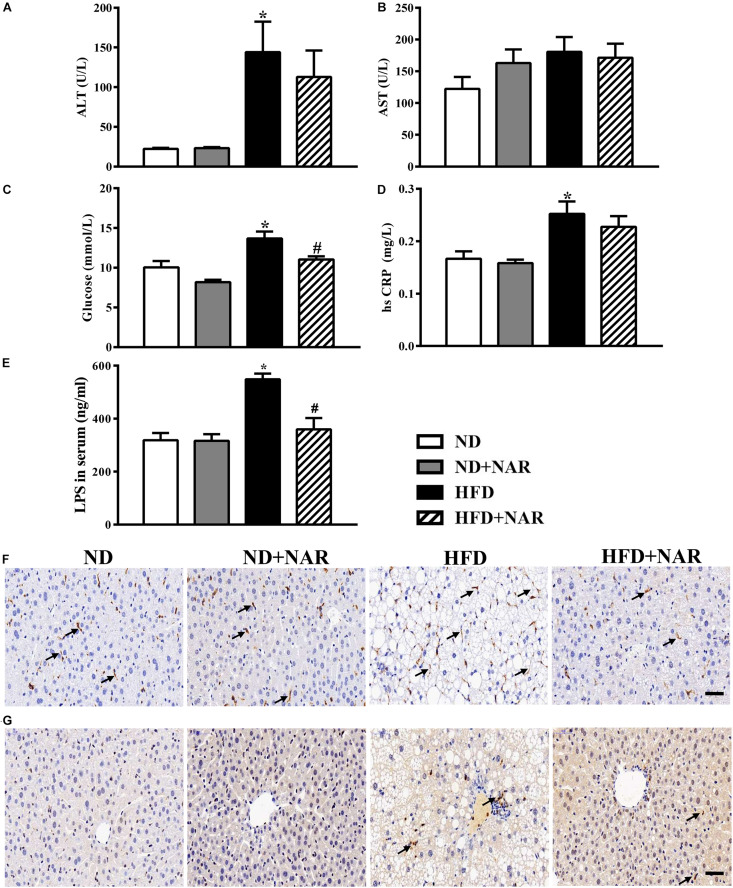
Effects of NAR on serum ALT, AST, glucose, hsCRP and LPS levels, and liver F4/80 and MPO in HFD-fed mice. Serum levels of **(A)** ALT, **(B)** AST, **(C)** glucose, **(D)** hsCRP, and **(E)** LPS. Liver samples in four groups stained by **(F)** F4/80 and **(G)** MPO. Bar = 50 μm. The arrows in **(F)** indicate the F4/80-positive cells. The arrows in **(G)** indicate the MPO-positive cells. Data are the mean ± SEM (*n* = 8). *P < 0.05 vs. ND group; #*P* < 0.05 vs. HFD group.

ALT and AST are markers of liver injury. Compared with the ND group, serum ALT levels were increased, and ALT levels were decreased in the NAR-treated HFD-fed mice (Figure 3A). We next measured the serum glucose and hsCRP, and HFD feeding enhanced the serum glucose and hsCRP production, while NAR supplementation only restored glucose in the serum (Figures 3C,D). HFD induced an LPS release in the serum, and increased F4/80-positive and MPO-positive cells in the liver, and NAR- treated HFD-fed mice displayed decreased LPS release in the serum and inflammatory cells in the liver (Figures 3E–G). Serum AST levels did not differ among the groups (Figure 3B).

### NAR Supplementation Reduced Lipogenesis

**FIGURE 4:**
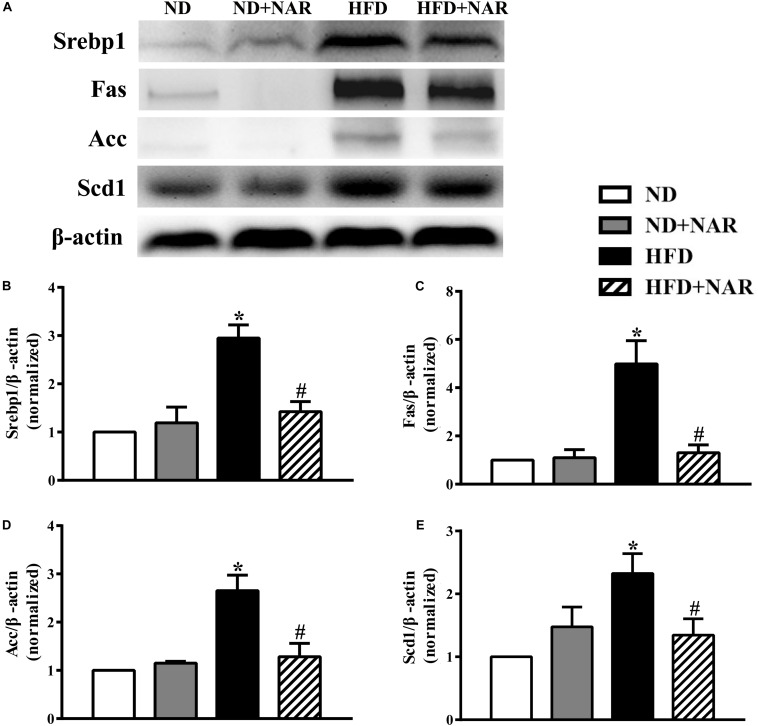
Effects of NAR on liver lipogenesis-related protein expression in HFD-fed mice. **(A)** Srebp1, Fas, Acc, and Scd1 expressions determined by western blot. **(B)** Quantitative analysis of Srebp1. **(C)** Quantitative analysis of Fas. **(D)** Quantitative analysis of Acc. **(E)** Quantitative analysis of Scd1. The blot image is representative of the four groups. The densitometry is an averaged result for the five animals, normalized to β-actin. Data are the mean ± SEM. *P < 0.05 vs. ND group; #*P* < 0.05 vs. HFD group.

To determine the mechanisms of the effects of NAR on lipid accumulation, we performed western blot analysis. HFD-fed mice had higher expression levels of key proteins involved in lipid metabolism (i.e., Srebp1, Fas, Acc, and Scd1) than did ND-fed mice (Figure 4). Notably, NAR treatment reduced the expressions of proteins involved in HFD-induced lipid metabolism in the liver tissue.

### NAR Treatment Altered the Gut Bacteria Composition in HFD-Induced Mice

The gut bacteria is thought to play a causal role in the NAFLD pathogenesis. We assessed the effects of NAR on the bacteria compositions in the ileum, cecum and colon using high-throughput sequencing of the bacterial 16S rRNA V3+V4 region.

In total, 14,987,642 raw reads were produced by high-throughput pyrosequencing of the samples. After removing the low-quality sequences, 14,531,807 clean tags were analyzed. Based on a 97% similarity level, all effective reads were clustered into OTUs.

**FIGURE 5:**
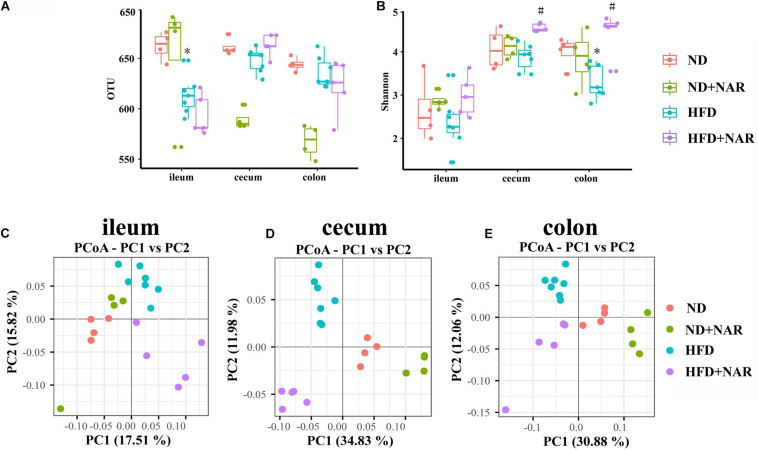
Effects of NAR on gut bacteria OTUs, diversity and similarity of species diversity. **(A)** OTU numbers of the gut bacteria from different parts of the gut in the four groups. **(B)** Alpha diversity of the gut bacteria assessed by the Shannon index for each group. PCoA analysis based on binary Jaccard distance algorithm of the gut bacteria in the **(C)** ileum, **(D)** cecum, and **(E)** colon. *P < 0.05 vs. ND group; #*P* < 0.05 vs. HFD group.

The rarefaction curves (Supplementary Figure S1) for all samples were flat with long tails, suggesting that most OTUs were included, and sufficient data were obtained. The observed OTUs and Chao1 and Shannon indices were used to show alpha diversities. The bacteria compositions from different parts of the gut showed varied performances in the mice even within the same group. The observed OTUs of the gut bacteria from different parts of the gut did not differ among the four groups, except that OTUs from the ileum, cecum and colon were decreased in the HFD group, compared with those of the ND group (Figure 5A). Interestingly, NAR supplementation decreased the cecal and colonic OTUs in ND-fed mice. ACE and Chao1 alpha diversity analyses revealed no differences in gut bacteria species richness between the groups (Supplementary Figures S2A,B). Shannon diversity results showed that HFD feeding decreased the colonic bacterial diversity, whereas NAR supplementation increased the diversity in the cecum and colon (Figure 5B). HFD feeding and NAR supplementation did not affect the ileal bacterial diversity.

Beta diversity analysis by binary Jaccard distance-based principal coordinate analysis (PCoA), based on OTU abundance was conducted to provide an overview of the extent of the similarities among the gut bacteria compositions after the different treatments. PCoA indicated distinct clustering of bacteria compositions in the ileum, cecum and colon for each treatment group (Figures 5C–E).

**FIGURE 6:**
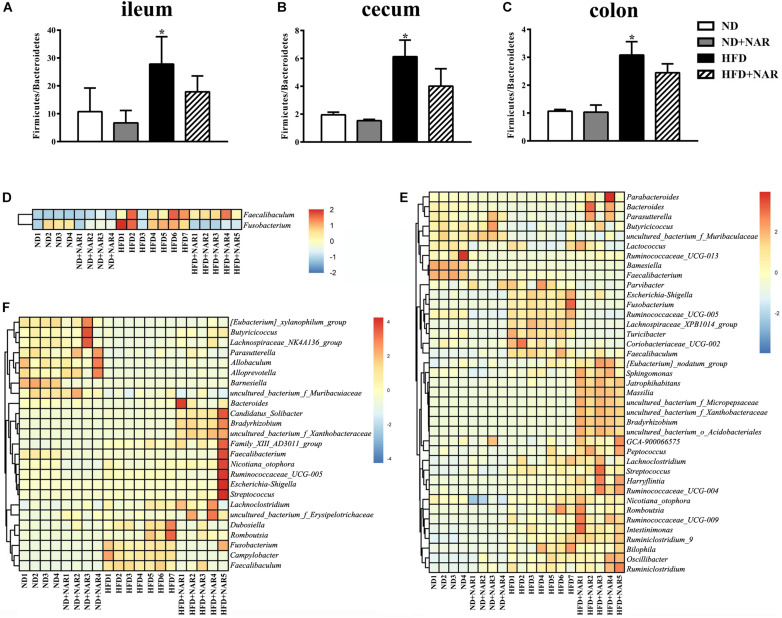
Effects of NAR on gut bacteria composition. Relative abundances of Firmicutes/Bacteroidetes in the **(A)** ileum, **(B)** cecum, and **(C)** colon. *P < 0.05 vs. ND group. Heatmaps of the gut bacteria compositions (those with mean relative abundances ≥0.05%) and by comparison (ND vs. ND+NAR, ND vs. HFD, and HFD vs. HFD+NAR, *q* ≤ 0.05 after correcting for the *p*-value) in the **(D)** ileum, **(E)** cecum, and **(F)** colon at the genus level.

We further assessed which gut microorganisms positively or negatively affected NAFLD development and progression. Although the different treatments showed no effects at the phylum level of the microflora (data not shown), HFD intervention increased the Firmicutes/Bacteroidetes ratio compared with that of the ND, while NAR increased this ratio in the HFD+NAR group (Figures 6A–C). Hence, NAR influenced the intestinal flora composition.

**FIGURE 7:**
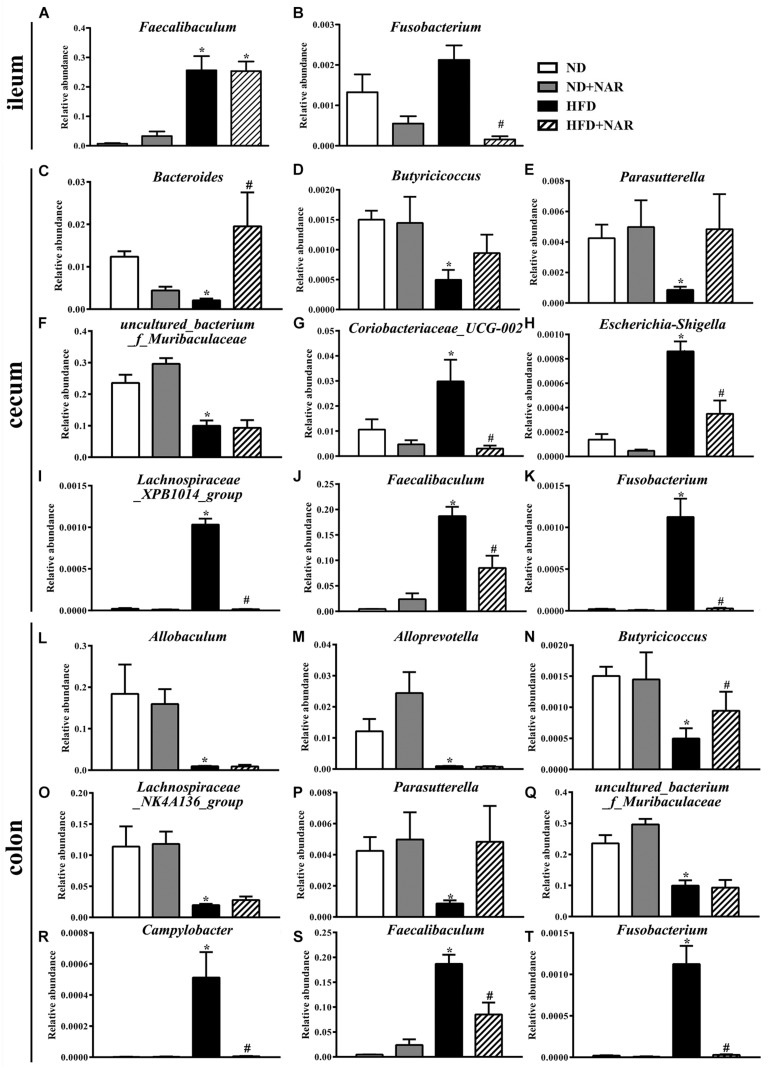
Effects of NAR on the relative abundances of several gut bacteria. Relative abundances of **(A)** *Faecalibaculum* and **(B)** *Fusobacterium* in the ileum, **(C)** *Bacteroides*, **(D)** *Butyricicoccus*, **(E)** *Parasutterella*, **(F)** *uncultured_bacterium_f_Muribaculaceae*, **(G)** *Coriobacteriaceae_UCG-002*, **(H)** *Escherichia-Shigella*, **(I)** *Lachnospiraceae_XPB1014_group*, **(J)** *Faecalibaculum and* **(K)** *Fusobacterium* in the cecum and **(L)** *Allobaculum*, **(M)** *Alloprevotella*, **(N)** *Butyricicoccus*, **(O)** *Lachnospiraceae_NK4A136_group*, **(P)** *Parasutterella*, **(Q)** *uncultured_bacterium_f_Muribaculaceae*, **(R)** *Campylobacter*, **(S)** *Faecalibaculum* and **(T)** *Fusobacterium* in the colon at the genus level, which in the HFD and HFD+NAR groups differed from ND group are presented. *P < 0.05 vs. ND group; #*P* < 0.05 vs. HFD group.

Heatmaps of dominant (mean relative abundance ≥0.05%) and different (ND vs. ND+NAR, ND vs. HFD, HFD vs. HFD+NAR, *q* ≤ 0.05 after correcting for the *p*-value, Supplementary Materials) genera strongly demonstrated that NAR supplementation reshaped HFD-induced changes in the gut bacteria profile (Figures 6D–F). The HFD group showed higher relative abundances of *Coriobacteriaceae_UCG-002*, *Escherichia-Shigella*, *Faecalibaculum*, *Fusobacterium*, *Lachnospiraceae_XPB1014_group*, *Parvibacter*, *Ruminococcaceae_UCG-005* and *Turicibacter* in the cecum and *Campylobacter*, *Dubosiella*, *Faecalibaculum* and *Fusobacterium* in the colon and a lower relative abundance of *Bacteroides* in the cecum and *Butyricicoccus* in the colon, compared with those of the ND group. NAR treatment reversed all of these effects. In addition, the gut communities of the HFD-fed mice showed lower relative abundances of *Butyricicoccus*, *Parasutterella* and *uncultured_bacterium_f_Muribaculaceae* in the cecum and *Allobaculum*, *Alloprevotella*, *Lachnospiraceae_NK4A136_group*, *Parasutterella* and *uncultured_bacterium_f_Muribaculaceae* in the colon and a higher relative abundance of *Faecalibaculum* in the ileum compared with those in the ND group. Interestingly, NAR supplementation in the HFD group induced higher relative abundances of *Bradyrhizobium*, *[Eubacterium]_nodatum_group*, *GCA-900066575*, *Jatrophihabitans*, *Massilia*, *Peptococcus*, *Sphingomonas*, *uncultured_bacterium_o_Acidobacteriales*, *uncultured_ bacterium_f_Micropepsaceae* and *uncultured_bacterium_f_ Xanthobacteraceae* in the cecum and *Bacteroides*, *Bradyrhizobium*, *Candidatus_Solibacter* and *uncultured_bacterium_ f_Xanthobacteraceae* in the colon and a lower relative abundance of *Fusobacterium* in the ileum. Figure 7 shows the statistical results for the bacteria whose relative abundances were altered. These data show that NAR modulated the gut bacteria of HFD-fed mice.

### Correlation Between the Gut Bacteria and Lipid Profile

**FIGURE 8:**
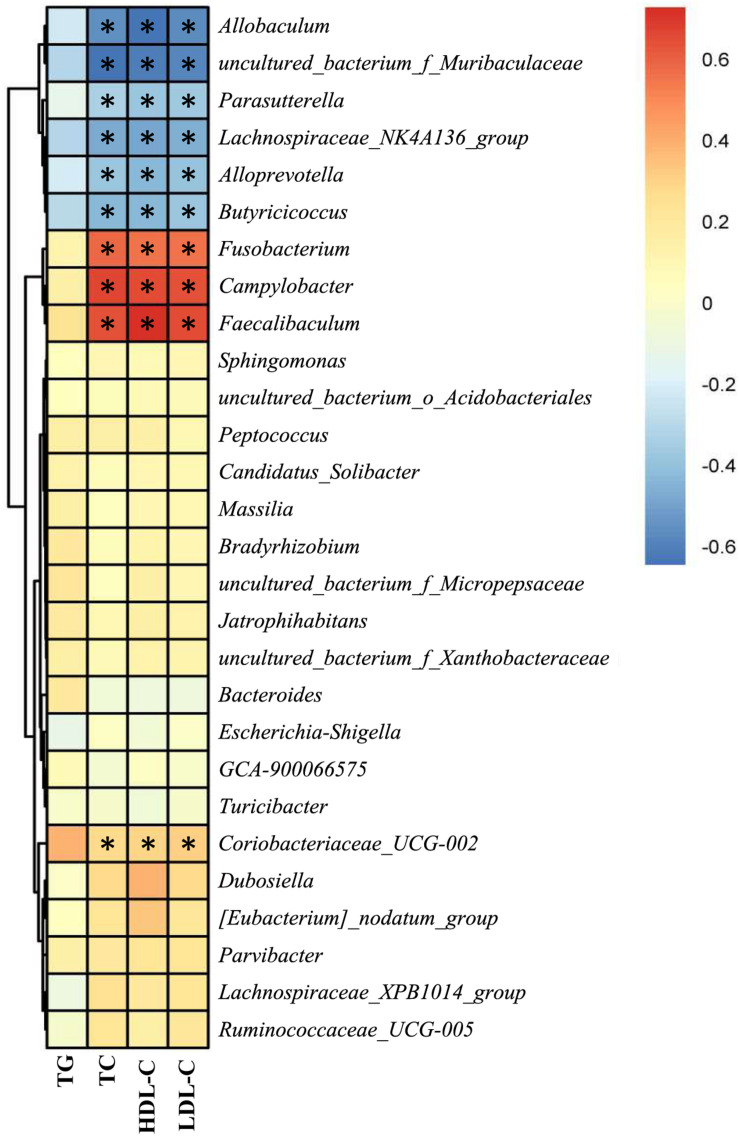
Correlation between the gut bacteria relative abundance and serum lipid level. Spearman rank correlations between altered gut bacteria and serum TG, TC, HDL-C, and LDL-C levels. Red indicates a positive correlation; blue indicates a negative correlation. Significant correlations are indicated by *P < 0.05 and marked with asterisks.

As NAR altered the gut bacteria compositions and improved serum lipid levels in HFD-fed mice, we analyzed the correlation between the above filtered fecal bacteria and serum lipid levels using Spearman rank correlation. Serum TC, HDL-C, and LDL-C levels were correlated with the relative abundances of the bacterial genera, among which *Allobaculum*, *Alloprevotella*, *Butyricicoccus*, *Lachnospiraceae_NK4A136_group*, *Parasutterella* and *uncultured_bacterium_f_Muribaculaceae* were negatively correlated with lipid levels, and *Campylobacter*, *Coriobacteriaceae_UCG-002*, *Faecalibaculum* and *Fusobacterium* were positively correlated with serum lipids (Figure 8).

## Discussion

In this study, we investigated the effect of NAR on NAFLD in HFD-fed mice. NAR reduced HFD- induced obesity, ameliorated the serum lipids, ALT, AST, glucose, hsCRP, and LPS levels, attenuated liver inflammation, blunted lipogenesis, and altered the gut bacteria compositions in NAFLD-model mice. In addition, the relative abundances at the genus level of *Allobaculum*, *Alloprevotella*, *Butyricicoccus*, *Lachnospiraceae_NK4A136_group*, *Parasutterella* and *uncultured_bacterium_f_Muribaculaceae* were negatively correlated with serum TC, HDL-C, and LDL-C levels, and the relative abundances of *Campylobacter*, *Coriobacteriaceae_UCG-002*, *Faecalibaculum*, and *Fusobacterium* were positively correlated with serum lipids. Thus, NAR may be a potential therapeutic adjuvant to improve NAFLD outcomes.

Non-alcoholic fatty liver disease is defined by abnormal lipid metabolism in the liver and is the most common liver disease worldwide (Yan et al., 2014; Ipsen et al., 2018; Lei et al., 2020). NAFLD is closely associated with cardiovascular diseases, which are a main cause of NAFLD-related deaths (Petta et al., 2015; Chang et al., 2019). Treating NAFLD remains a challenge, with no effective drugs available. NAR, a Chinese herbal medicine, is widely found in citrus plants and possesses great health benefits (Liu et al., 2020). NAR may be a potential treatment for NAFLD because of its antilipidemic effects (Raffoul-Orozco et al., 2018; Zhou et al., 2019), but how NAR acts on lipid accumulation remains uncertain.

The regulation of lipogenic gene expression is mainly mediated by transcription factors, among which, Srebp1 is a master regulator to most hepatic lipid synthesis genes, including Fas, Acc, and Scd1 (Guo et al., 2018; Zhou et al., 2020). Fas, provides a non-esterified fatty-acid substrate for triacylglycerol, resulting in enhanced fatty acid synthesis and TG accumulation (Yang et al., 2020). Acc converts acetyl-CoA to malonyl CoA, which participates in the synthesis of fatty acids (Li et al., 2020). Scd1 contributes to fatty acid desaturation (Sheng et al., 2019). Srebp1 and its target genes including Fas, Acc, and Scd1 are up-regulated in NAFLD, and inhibiting Srebp1 activity impairs the induction of lipogenic Srebp1 target genes and TG accumulation (Bitter et al., 2015a, b). To investigate the role of NAR in regulating hepatic *de novo* fatty acid synthesis, we detected the expression of several key transcriptional regulators in liver tissue: Srebp1, Fas, Acc, and Scd1. As expected, Srebp1, Fas, Acc, and Scd1 expressions were increased in the HFD group, and NAR efficiently attenuated hepatic *de novo* fatty acid synthesis by downregulating these proteins (Figure 4). This finding was consistent with the results illustrated in the liver morphology, histology, and blood lipids. Body weight, liver weight, liver/body weight and serum ALT, glucose and hsCRP were decreased in the HFD group after NAR treatment (Figures 1, 2, 3A–D).

The gut bacteria plays an active role in human physiology (Chi et al., 2019). Imbalances in the gut bacteria are associated with many chronic diseases, including NAFLD, obesity, diabetes, and colon cancer (Ridaura et al., 2013). Long-term dietary habits shape the bacteria composition and function; therefore, diet modifications to the gut bacteria could be a new therapeutic approach for treating NAFLD (Wu et al., 2011). Despite accumulating studies revealing an association between gut bacteria dysbiosis and NAFLD, the mechanisms of the gut dysbiosis that result in NAFLD injury remain unclear. The potential pathophysiology can be summarized as follows. (1) In promoting hepatic inflammation, patients with NAFLD normally have impaired gut barrier integrity, bacterial overgrowth, and bacterial translocation, which can help release LPS through the gut-liver axis, thus resulting in an inflammatory cascade, lipid accumulation and hepatocyte death, further disrupting the intestinal barrier and benefiting bacterial translocation (Mouries et al., 2019). HFD induced an LPS release in the serum and increased F4/80-positive and MPO-positive cells in the liver. NAR-treated HFD-fed mice displayed decreased LPS release in the serum and inflammatory cells in the liver (Figures 3E–G). Gut bacteria dysbiosis also plays a critical role in weakening the mucosal immunity in NAFLD hosts (Xie and Halegoua-Demarzio, 2019). (2) Altered biochemistry metabolism and gut bacteria-related metabolites, such as bile acid, short-chain fatty acids, aromatic amino acid derivatives, branched-chain amino acids, choline and ethanol, as well as disordered metabolism, exert metabolic and immunologic effects that contribute to NAFLD (Leung et al., 2016). (3) Regarding disrupting the balance between energy harvesting and expenditure, the gut bacteria possesses an enriched phosphotransferase system and alters the levels of metabolic products. This is why the gut bacteria is involved in energy influx and expenditure, which may contribute to NAFLD development (Turnbaugh et al., 2009; Broeders et al., 2015).

Natural compounds in the diet, such as polyphenols, can reduce hepatic lipid accumulation and modify the gut microbial balance, thus presenting great therapeutic potential for treating NAFLD (Porras et al., 2017; Fraga et al., 2019). Because of the recently reported function of polyphenols in regulating lipid accumulation and gut microbes, we explored the likely involvement of NAR in modulating the intestinal bacterial composition. Although the OTUs and alpha diversity analyses, including the ACE, Chao1, and Shannon indices, revealed different statistical results, the richness and diversity of the gut bacteria were decreased in HFD-fed mice. NAR treatment partly reversed this phenomenon (Figures 5A,B and Supplementary Figure S2). PCoA indicated separate bacteria between the ND group and the other treatment groups (Figures 5C–E). These data imply that changes in the gut bacteria might be partially responsible for the effective intervention of NAR on lipid accumulation in NAFLD-model mice.

To further identify the fecal bacteria community which was different from the ND mice, we analyzed the bacteria at the phylum and genus levels. At the phylum level, NAR supplementation decreased the Firmicutes/Bacteroidetes ratio in HFD-fed mice (Figures 6A–C). Consistently, several reports revealed that polyphenols and Chinese herbal extracts, such as *Nitzschia laevis* extract (Guo et al., 2019), *Citrus aurantium L. var. amara* Engl. (Shen et al., 2019) and a combination of quercetin and resveratrol (Zhao et al., 2017), decreased the Firmicutes/Bacteroidetes ratio, thus shifting the gut bacteria toward a healthy composition. An increased Firmicutes/Bacteroidetes ratio is a typical characteristic in obese humans and mammals. Because Firmicutes can produce more harvestable energy than can Bacteroidetes, the relative higher abundance of Firmicutes leads to increased calorie absorption and promotes obesity (Komaroff, 2017). In the current study, NAR treatment helped maintain the Firmicutes/Bacteroidetes ratio at a lower level, which might contribute to its antilipidemic effect.

At the genus level, *Allobaculum*, *Alloprevotella*, *Butyricicoccus*, *Lachnospiraceae_NK4A136_group*, *Parasutterella* and *uncultured_bacterium_f_Muribaculaceae* were obviously negatively correlated with serum lipid levels, and *Campylobacter*, *Coriobacteriaceae_UCG-002*, *Faecalibaculum* and *Fusobacterium* were positively correlated with serum lipid levels (Figure 8). Consistently, HFD-fed mice exhibited fewer *Allobaculum*, *Alloprevotella*, *Butyricicoccus*, *Lachnospiraceae_NK4A136_group*, *Parasutterella* and *uncultured_bacterium_f_Muribaculaceae* than did the ND group, while NAR treatment only reversed the relative abundance of *Butyricicoccus* in the colons of HFD-fed mice. Relative abundances of *Campylobacter*, *Coriobacteriaceae_UCG-002*, *Faecalibaculum* and *Fusobacterium* were increased in the HFD group, and NAR treatment reduced the relative abundances of these intestinal flora. HFD-fed mice also presented more *Dubosiella*, *Escherichia-Shigella*, *Lachnospiraceae_XPB1014_group*, *Parvibacter*, *Ruminococcaceae_UCG-005* and *Turicibacter* but fewer *Bacteroides*, which were mostly restored by NAR treatment. NAR supplementation in HFD-fed mice induced higher abundances of *Bradyrhizobium*, *Candidatus _ Solibacter*, *[Eubacterium]_nodatum_group*, *GCA-900066575*, *Jatrophihabitans*, *Massilia*, *Peptococcus*, *Sphingomonas*, *uncultured_bacterium_o_Acidobacteriales*, *uncultured_bacterium_f_Micropepsaceae* and *uncultured_bacterium_f_Xanthobacteraceae* (Figures 6D–F, 7). These altered bacteria may participate in NAFLD progression, and NAR intervention leads to structural modulation of the gut bacteria, which might help mitigate NAFLD.

Some bacteria, such as *Allobaculum*, *Alloprevotella* and *Bacteroides*, are reported to produce short-chain fatty acids and have potential anti-obesity activity (Berry et al., 2015; Fan et al., 2015; Wang et al., 2020b). The Lachnospiraceae family can produce or regulate butyrate to maintain the gut barrier integrity (Chen et al., 2019). *Lachnospiraceae_XPB1014_group* has been negatively correlated with body fat weight (Zhou et al., 2018), and *Lachnospiraceae_NK4A316_group* are harmful bacteria (Wang et al., 2020b). *Escherichia-Shigella*, *Faecalibaculum*, and *Fusobacterium* are proinflammatory bacteria that may impair the gut barrier (Yin et al., 2013; Neubauer et al., 2019; Cai et al., 2020) and are associated with exacerbated hepatic steatosis (Henao-Mejia et al., 2012). *Campylobacter* is an opportunistic pathogen that affects host health (Moon et al., 2018). Patients with NAFLD were reported to have increased levels of *Bradyrhizobium* (Del Chierico et al., 2017), and *Turicibacter* was positively correlated with lipid metabolism indicators (Li L. et al., 2019; Li T. T. et al., 2019); however, the detailed mechanism by which these bacteria are involved in NAFLD is unclear. Additionally, the effects on lipid metabolism of some bacteria with altered relative abundances, such as *Butyricicoccus*, *Candidatus _ Solibacter*, *Coriobacteriaceae_UCG-002*, *Dubosiella*, and *GCA-900066575*, are unclear, and these bacteria might also participate in lipid metabolism. Therefore, we conclude that NAR benefited the balance between lipid metabolism and prevention of NAFLD progression, likely by restoring specific gut microbes to a normal healthy baseline.

Naringin has low oral bioavailability and is poorly absorbed in the circulatory system. Therefore, orally administered NAR remains in the gastrointestinal tract for a relatively long time, and gut microbes would be a crucial target of NAR *in vivo*. Studies of the detailed metabolic processes of NAR have been conducted in humans (Chen et al., 2018), rats (Zeng et al., 2020), and mice (Orrego-Lagaron et al., 2015). Thirteen human microbial metabolites have been detected and identified (Chen et al., 2018). When orally administered, lactase-phlorizin hydrolase and the intestinal microflora hydrolyze NAR to its aglycon, naringenin (Chen et al., 2014). Naringenin is partly absorbed, then engaged in both phase I and phase II metabolism. Mediated by the gut bacteria, unabsorbed naringenin and metabolites from the bile are further catabolized into phenolic products such as 3-(4′-hydroxyphenyl)propionic acid (HPPA), 4′-hydroxybenzoic acid, and hippuric acid (Zeng et al., 2019, 2020). Naringenin and HPPA are the major microbial metabolites. Naringenin attenuates NAFLD by reducing inflammation, lipoprotein metabolism, and dyslipidemia (Mulvihill et al., 2016; Wang et al., 2020a). HPPA can effectively suppress influenza (Steed et al., 2017). Intestinal microbe-mediated metabolism may play an important role in regulating both the pharmacokinetics and bioactive properties of NAR; NAR simultaneously modulated the gut bacteria composition and influenced bacterial growth.

Goblet cells are specialized for mucus synthesis and secretion; hence, goblet cells play an important role in maintaining gut permeability (Birchenough et al., 2015), and gut permeability, endotoxemia, inflammation and gut bacteria dysbiosis are tightly connected. In HFD-fed rats, mucosal layer thickness is markedly reduced, goblet cells are overgrown, and the gut flora is dysregulated, leading to increased intestinal permeability, which eventually promotes the development of metabolic endotoxemia, inflammation and metabolic disorders. Thus, inhibition overgrown of goblet cells, may modulate gut permeability, microbial dysbiosis in HFD-fed rats and exert health benefits. In this study, we fed mice HFD supplemented with NAR and found that NAR supplementation attenuated NAFLD parameters in the HFD-fed mice. NAR also altered the community compositional structure of the gut bacteria characterized by increased beneficial bacteria and fewer harmful bacteria. Thus, NAR may help attenuate NAFLD by preventing gut dysbiosis; however, this was not confirmed. The present study lacked experimental evidence to confirm that NAR could attenuate NAFLD by directly modulating the gut bacteria. In addition, given the important role of goblet cells in controlling intestinal permeability, further experiments on mouse goblet cells are necessary to explore the NAR mechanism of action.

## Conclusion

**FIGURE 9:**
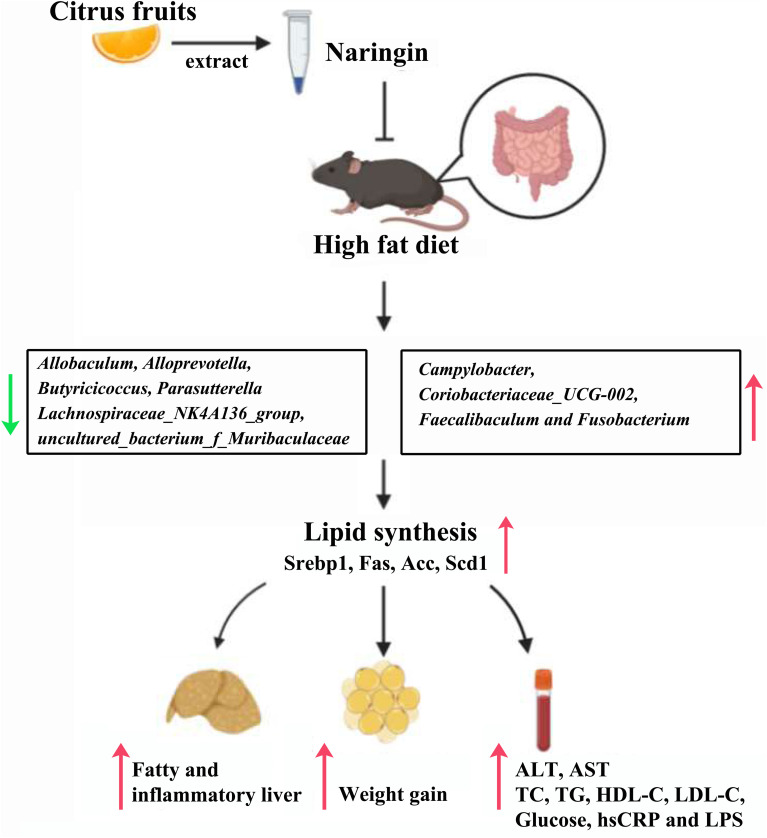
Proposed mechanisms for protective effects of NAR on HFD-fed mice. NAR reduces lipid synthesis and attenuates NAFLD parameters, possibly by modulating the gut bacteria composition via an unknown pathway.

The present study demonstrated the effectiveness of treatment with NAR on gut bacteria in NAFLD mice for the first time. In this study, we found NAR altered the community compositional structure of gut bacteria, and attenuated NAFLD parameters, in addition, we demonstrated that the relative abundances of some bacteria were closely related to serum lipid levels, thus, NAR may protect against HFD-induced liver damage by modulating the gut bacteria composition via an unknown pathway (Figure 9).

## Data Availability Statement

The original contributions presented in the study are publicly available. This data can be found in the Genome Sequence Archive (GSA), under accession CRA003374.

## Ethics Statement

The animal study was reviewed and approved by the Peking University Biomedical Ethics Committee Experimental Animal Ethics Branch.

## Author Contributions

HM and JD designed the research and drafted the article. QZ performed the gut bacteria analysis and processed the data. RY and JZ performed the biochemical assays and bred the animals. XL and RZ performed the histological analysis and bred the animals. WT and HL performed the western blotting assay. SW, TS, XH, and LD revised the manuscript. All authors read and approved the final manuscript.

## Conflict of Interest

The authors declare that the research was conducted in the absence of any commercial or financial relationships that could be construed as a potential conflict of interest.

## Abbreviations

Acc: acetyl-CoA carboxylase 1
ALT: alanine aminotransferase
AST: aspartate aminotransferase
Fas: fatty acid synthase
HDL-C: high-density lipoprotein cholesterol
HE: hematoxylin and eosin
HFD: high-fat diet
hsCRP: high-sensitivity C-reactive protein
LDL-C: low-density lipoprotein cholesterol
LPS: lipopolysaccharides
MPO: myeloperoxidase
NAR: naringin
NAFLD: non-alcoholic fatty liver disease
ND: normal diet
Scd1: stearoyl-CoA desaturase 1
OTU: operational taxonomic units
Srebp1: sterol regulatory element binding protein1
TC: total cholesterol
TG: triglycerides.

## References

[B1] Ahmed O. M. Hassan M. A. Abdel-Twab S. M. Abdel Azeem M. N. (2017). Navel orange peel hydroethanolic extract, naringin and naringenin have anti-diabetic potentials in type 2 diabetic rats. *Biomed. Pharmacother.* 94 197–205. 10.1016/j.biopha.2017.07.094 28759757

[B2] Aron-Wisnewsky J. Vigliotti C. Witjes J. Le P. Holleboom A. G. Verheij J. (2020). Gut microbiota and human NAFLD: disentangling microbial signatures from metabolic disorders. *Nat. Rev. Gastroenterol. Hepatol.* 17 279–297. 10.1038/s41575-020-0269-9 32152478

[B3] Berry D. Mader E. Lee T. K. Woebken D. Wang Y. Zhu D. (2015). Tracking heavy water (D2O) incorporation for identifying and sorting active microbial cells. *Proc. Natl. Acad. Sci. U.S.A.* 112 E194–E203. 10.1073/pnas.1420406112 25550518 PMC4299247

[B4] Birchenough G. M. Johansson M. E. Gustafsson J. K. Bergstrom J. H. Hansson G. C. (2015). New developments in goblet cell mucus secretion and function. *Mucosal. Immunol.* 8 712–719. 10.1038/mi.2015.32 25872481 PMC4631840

[B5] Bitter A. Nüssler A. K. Thasler W. E. Klein K. Zanger U. M. Schwab M. (2015a). Human sterol regulatory element-binding protein 1a contributes significantly to hepatic lipogenic gene expression. *Cell Physiol. Biochem.* 35 803–815. 10.1159/000369739 25634759

[B6] Bitter A. Rümmele P. Klein K. Kandel B. A. Rieger J. K. Nüssler A. K. (2015b). Pregnane X receptor activation and silencing promote steatosis of human hepatic cells by distinct lipogenic mechanisms. *Arch. Toxicol.* 89 2089–2103. 10.1007/s00204-014-1348-x 25182422

[B7] Bolger A. M. Lohse M. Usadel B. (2014). Trimmomatic: a flexible trimmer for Illumina sequence data. *Bioinformatics* 30, 2114–2120. 10.1093/bioinformatics/btu170 24695404 PMC4103590

[B8] Boursier J. Mueller O. Barret M. Machado M. Fizanne L. Araujo-Perez F. (2016). The severity of nonalcoholic fatty liver disease is associated with gut dysbiosis and shift in the metabolic function of the gut microbiota. *Hepatology* 63 764–775. 10.1002/hep.28356 26600078 PMC4975935

[B9] Broeders E. P. Nascimento E. B. Havekes B. Brans B. Roumans K. H. Tailleux A. (2015). The Bile Acid Chenodeoxycholic Acid Increases Human Brown Adipose Tissue Activity. *Cell Metab.* 22 418–426. 10.1016/j.cmet.2015.07.002 26235421

[B10] Cai W. Xu J. Li G. Liu T. Guo X. Wang H. (2020). Ethanol extract of propolis prevents high-fat diet-induced insulin resistance and obesity in association with modulation of gut microbiota in mice. *Food Res. Int.* 130:108939. 10.1016/j.foodres.2019.108939 32156386

[B11] Chang Y. Ryu S. Sung K. C. Cho Y. K. Sung E. Kim H. N. (2019). Alcoholic and non-alcoholic fatty liver disease and associations with coronary artery calcification: evidence from the Kangbuk Samsung Health Study. *Gut* 68 1667–1675. 10.1136/gutjnl-2018-317666 30472683

[B12] Chen R. Wu P. Cai Z. Fang Y. Zhou H. Lasanajak Y. (2019). Puerariae lobatae radix with chuanxiong Rhizoma for treatment of cerebral ischemic stroke by remodeling gut microbiota to regulate the brain-gut barriers. *J. Nutr. Biochem.* 65 101–114. 10.1016/j.jnutbio.2018.12.004 30710886

[B13] Chen T. Su W. Yan Z. Wu H. Zeng X. Peng W. (2018). Identification of naringin metabolites mediated by human intestinal microbes with stable isotope-labeling method and UFLC-Q-TOF-MS/MS. *J. Pharm. Biomed. Anal.* 161 262–272. 10.1016/j.jpba.2018.08.039 30172881

[B14] Chen Z. Zheng S. Li L. Jiang H. (2014). Metabolism of flavonoids in human: a comprehensive review. *Curr. Drug. Metab.* 15 48–61. 10.2174/138920021501140218125020 24588554

[B15] Chi Y. Lin Y. Lu Y. Huang Q. Ye G. Dong S. (2019). Gut microbiota dysbiosis correlates with a low-dose PCB126-induced dyslipidemia and non-alcoholic fatty liver disease. *Sci. Total Environ.* 653 274–282. 10.1016/j.scitotenv.2018.10.387 30412872

[B16] Del Chierico F. Nobili V. Vernocchi P. Russo A. De Stefanis C. Gnani D. (2017). Gut microbiota profiling of pediatric nonalcoholic fatty liver disease and obese patients unveiled by an integrated meta-omics-based approach. *Hepatology* 65 451–464. 10.1002/hep.28572 27028797

[B17] Fan P. Li L. Rezaei A. Eslamfam S. Che D. Ma X. (2015). Metabolites of dietary protein and peptides by intestinal microbes and their Impacts on Gut. *Curr. Protein Pept. Sci.* 16 646–654. 10.2174/1389203716666150630133657 26122784

[B18] Fraga C. G. Croft K. D. Kennedy D. O. Tomas-Barberan F. A. (2019). The effects of polyphenols and other bioactives on human health. *Food Funct.* 10 514–528. 10.1039/c8fo01997e 30746536

[B19] Fu D. Cui H. Zhang Y. (2018). Lack of ClC-2 alleviates high fat diet-induced insulin resistance and non-alcoholic fatty liver disease. *Cell Physiol. Biochem.* 45 2187–2198. 10.1159/000488164 29550812

[B20] Glass O. Filozof C. Noureddin M. Berner-Hansen M. Schabel E. Omokaro S. O. (2020). Standardization of diet and exercise in clinical trials of Nafld-Nash: recommendations from the liver forum. *J. Hepatol.* 73 680–693. 10.1016/j.jhep.2020.04.030 32353483

[B21] Guo B. Liu B. Wei H. Cheng K. W. Chen F. (2019). Extract of the microalga nitzschia laevis prevents high-fat-diet-induced obesity in mice by modulating the composition of gut microbiota. *Mol. Nutr. Food Res.* 63 e1800808. 10.1002/mnfr.201800808 30475446

[B22] Guo J. Fang W. Sun L. Lu Y. Dou L. Huang X. (2018). Ultraconserved element uc.372 drives hepatic lipid accumulation by suppressing miR-195/miR4668 maturation. *Nat. Commun.* 9:612. 10.1038/s41467-018-03072-8 29426937 PMC5807361

[B23] Henao-Mejia J. Elinav E. Jin C. Hao L. Mehal W. Z. Strowig T. (2012). Inflammasome-mediated dysbiosis regulates progression of NAFLD and obesity. *Nature* 482 179–185. 10.1038/nature10809 22297845 PMC3276682

[B24] Ipsen D. H. Lykkesfeldt J. Tveden-Nyborg P. (2018). Molecular mechanisms of hepatic lipid accumulation in non-alcoholic fatty liver disease. *Cell Mol. Life Sci.* 75 3313–3327. 10.1007/s00018-018-2860-6 29936596 PMC6105174

[B25] Komaroff A. L. (2017). The microbiome and risk for obesity and diabetes. *JAMA* 317 355–356. 10.1001/jama.2016.20099 28006047

[B26] Le Roy T. Llopis M. Lepage P. Bruneau A. Rabot S. Bevilacqua C. (2013). Intestinal microbiota determines development of non-alcoholic fatty liver disease in mice. *Gut* 62 1787–1794. 10.1136/gutjnl-2012-303816 23197411

[B27] Lei Y. Hoogerland J. A. Bloks V. W. Bos T. Bleeker A. Wolters H. (2020). Hepatic ChREBP activation limits NAFLD development in a mouse model for glycogen storage disease type Ia. *Hepatology* 10.1002/hep.31198 Online ahead of print 32083759 PMC7702155

[B28] Leung C. Rivera L. Furness J. B. Angus P. W. (2016). The role of the gut microbiota in NAFLD. *Nat. Rev. Gastroenterol. Hepatol.* 13 412–425. 10.1038/nrgastro.2016.85 27273168

[B29] Li L. Guo W. L. Zhang W. Xu J. X. Qian M. Bai W. D. (2019). Grifola frondosa polysaccharides ameliorate lipid metabolic disorders and gut microbiota dysbiosis in high-fat diet fed rats. *Food Funct.* 10 2560–2572. 10.1039/c9fo00075e 30994668

[B30] Li T. T. Tong A. J. Liu Y. Y. Huang Z. R. Wan X. Z. Pan Y. Y. (2019). Polyunsaturated fatty acids from microalgae Spirulina platensis modulates lipid metabolism disorders and gut microbiota in high-fat diet rats. *Food Chem. Toxicol.* 131:110558. 10.1016/j.fct.2019.06.005 31175915

[B31] Li T. Li X. Meng H. Chen L. Meng F. (2020). ACSL1 affects triglyceride levels through the PPARγ pathway. *Int. J. Med. Sci.* 17 720–727. 10.7150/ijms.42248 32218693 PMC7085263

[B32] Lima A. C. D. Cecatti C. Fidelix M. P. Adorno M. A. T. Sakamoto I. K. Cesar T. B. (2019). Effect of daily consumption of orange juice on the levels of blood glucose, lipids, and gut microbiota metabolites: controlled clinical trials. *J. Med. Food* 22 202–210. 10.1089/jmf.2018.0080 30638420

[B33] Liu P. Bian Y. Fan Y. Zhong J. Liu Z. (2020). Protective effect of naringin on in vitro gut-vascular barrier disruption of intestinal microvascular endothelial cells induced by TNF-alpha. *J. Agric. Food Chem.* 68 168–175. 10.1021/acs.jafc.9b06347 31850758

[B34] Magoč T. Salzberg S. L. (2011). FLASH: fast length adjustment of short reads to improve genome assemblies. *Bioinformatics* 27, 2957–2963. 10.1093/bioinformatics/btr507 21903629 PMC3198573

[B35] Moon C. D. Young W. Maclean P. H. Cookson A. L. Bermingham E. N. (2018). Metagenomic insights into the roles of *Proteobacteria* in the gastrointestinal microbiomes of healthy dogs and cats. *Microbiologyopen* 7:e00677. 10.1002/mbo3.677 29911322 PMC6182564

[B36] Mouries J. Brescia P. Silvestri A. Spadoni I. Sorribas M. Wiest R. (2019). Microbiota-driven gut vascular barrier disruption is a prerequisite for non-alcoholic steatohepatitis development. *J. Hepatol.* 71 1216–1228. 10.1016/j.jhep.2019.08.005 31419514 PMC6880766

[B37] Mulvihill E. E. Burke A. C. Huff M. W. (2016). Citrus flavonoids as regulators of lipoprotein metabolism and atherosclerosis. *Annu. Rev. Nutr.* 36 275–299. 10.1146/annurev-nutr-071715-50718 27146015

[B38] Neubauer V. Humer E. Mann E. Kroger I. Reisinger N. Wagner M. (2019). Effects of clay mineral supplementation on particle-associated and epimural microbiota, and gene expression in the rumen of cows fed high-concentrate diet. *Anaerobe* 59 38–48. 10.1016/j.anaerobe.2019.05.003 31102775

[B39] Orrego-Lagaron N. Martinez-Huelamo M. Vallverdu-Queralt A. Lamuela-Raventos R. M. Escribano-Ferrer E. (2015). High gastrointestinal permeability and local metabolism of naringenin: influence of antibiotic treatment on absorption and metabolism. *Br. J. Nutr.* 114 169–180. 10.1017/S0007114515001671 26083965

[B40] Pais R. Barritt A. S. T. Calmus Y. Scatton O. Runge T. Lebray P. (2016). NAFLD and liver transplantation: current burden and expected challenges. *J. Hepatol.* 65 1245–1257. 10.1016/j.jhep.2016.07.033 27486010 PMC5326676

[B41] Petta S. Argano C. Colomba D. Camma C. Di Marco V. Cabibi D. (2015). Epicardial fat, cardiac geometry and cardiac function in patients with non-alcoholic fatty liver disease: association with the severity of liver disease. *J. Hepatol.* 62 928–933. 10.1016/j.jhep.2014.11.030 25445395

[B42] Porras D. Nistal E. Martinez-Florez S. Pisonero-Vaquero S. Olcoz J. L. Jover R. (2017). Protective effect of quercetin on high-fat diet-induced non-alcoholic fatty liver disease in mice is mediated by modulating intestinal microbiota imbalance and related gut-liver axis activation. *Free Radic. Biol. Med.* 102 188–202. 10.1016/j.freeradbiomed.2016.11.037 27890642

[B43] Raffoul-Orozco A. K. Avila-Gonzalez A. E. Rodriguez-Razon C. M. Garcia-Cobian T. A. Perez-Guerrero E. E. Garcia-Iglesias T. (2018). Combination effect naringin and pravastatin in lipid profile and glucose in obese rats. *Life Sci.* 193 87–92. 10.1016/j.lfs.2017.11.044 29197498

[B44] Ridaura V. K. Faith J. J. Rey F. E. Cheng J. Duncan A. E. Kau A. L. (2013). Gut microbiota from twins discordant for obesity modulate metabolism in mice. *Science* 341:1241214. 10.1126/science.1241214 24009397 PMC3829625

[B45] Rotimi S. O. Adelani I. B. Bankole G. E. Rotimi O. A. (2018). Naringin enhances reverse cholesterol transport in high fat/low streptozocin induced diabetic rats. *Biomed. Pharmacother.* 101 430–437. 10.1016/j.biopha.2018.02.116 29501765

[B46] Schoeler M. Caesar R. (2019). Dietary lipids, gut microbiota and lipid metabolism. *Rev. Endocr. Metab. Disord.* 20 461–472. 10.1007/s11154-019-09512-0 31707624 PMC6938793

[B47] Shen C. Y. Wan L. Wang T. X. Jiang J. G. (2019). Citrus aurantium L. var. amara Engl. inhibited lipid accumulation in 3T3-L1 cells and *Caenorhabditis elegans* and prevented obesity in high-fat diet-fed mice. *Pharmacol. Res.* 147:104347. 10.1016/j.phrs.2019.104347 31315066

[B48] Sheng D. Zhao S. Gao L. Zheng H. Liu W. Hou J. (2019). BabaoDan attenuates high-fat diet-induced non-alcoholic fatty liver disease via activation of AMPK signaling. *Cell Biosci.* 9:77. 10.1186/s13578-019-0339-2 31548878 PMC6751621

[B49] Steed A. L. Christophi G. P. Kaiko G. E. Sun L. Goodwin V. M. Jain U. (2017). The microbial metabolite desaminotyrosine protects from influenza through type I interferon. *Science* 357 498–502. 10.1126/science.aam5336 28774928 PMC5753406

[B50] Sui G. G. Xiao H. B. (2018). Naringin activates AMPK resulting in altered expression of SREBPs, PCSK9, and LDLR to reduce body weight in obese C57BL/6J mice. *J. Agric. Food Chem.* 66 8983–8990. 10.1021/acs.jafc.8b02696 30092639

[B51] Turnbaugh P. J. Backhed F. Fulton L. Gordon J. I. (2008). Diet-induced obesity is linked to marked but reversible alterations in the mouse distal gut microbiome. *Cell Host. Microbe* 3 213–223. 10.1016/j.chom.2008.02.015 18407065 PMC3687783

[B52] Turnbaugh P. J. Hamady M. Yatsunenko T. Cantarel B. L. Duncan A. Ley R. E. (2009). A core gut microbiome in obese and lean twins. *Nature* 457 480–484. 10.1038/nature07540 19043404 PMC2677729

[B53] Wang D. Yan J. Chen J. Wu W. Zhu X. Wang Y. (2015). Naringin improves neuronal insulin signaling, brain mitochondrial function, and cognitive function in high-fat diet-induced obese mice. *Cell Mol. Neurobiol.* 35 1061–1071. 10.1007/s10571-015-0201-y 25939427 PMC11486290

[B54] Wang P. Wang J. Li D. Ke W. Chen F. Hu X. (2020a). Targeting the gut microbiota with resveratrol: a demonstration of novel evidence for the management of hepatic steatosis. *J. Nutr. Biochem.* 81:108363. 10.1016/j.jnutbio.2020.108363 32388250

[B55] Wang Q. Ou Y. Hu G. Wen C. Yue S. Chen C. (2020b). Naringenin attenuates non-alcoholic fatty liver disease by down-regulating the NLRP3/NF-kappaB pathway in mice. *Br. J. Pharmacol.* 177 1806–1821. 10.1111/bph.14938 31758699 PMC7070172

[B56] Wu G. D. Chen J. Hoffmann C. Bittinger K. Chen Y. Y. Keilbaugh S. A. (2011). Linking long-term dietary patterns with gut microbial enterotypes. *Science* 334 105–108. 10.1126/science.1208344 21885731 PMC3368382

[B57] Xie C. Halegoua-Demarzio D. (2019). Role of probiotics in non-alcoholic fatty liver disease: does gut microbiota matter? *Nutrients* 11:2837. 10.3390/nu11112837 31752378 PMC6893593

[B58] Yan F. Wang Q. Lu M. Chen W. Song Y. Jing F. (2014). Thyrotropin increases hepatic triglyceride content through upregulation of SREBP-1c activity. *J. Hepatol.* 61 1358–1364. 10.1016/j.jhep.2014.06.037 25016220

[B59] Yang C. Xu Z. Deng Q. Huang Q. Wang X. Huang F. (2020). Beneficial effects of flaxseed polysaccharides on metabolic syndrome via gut microbiota in high-fat diet fed mice. *Food Res. Int.* 131:108994. 10.1016/j.foodres.2020.108994 32247451

[B60] Yin J. Li Y. Han H. Chen S. Gao J. Liu G. (2018). Melatonin reprogramming of gut microbiota improves lipid dysmetabolism in high-fat diet-fed mice. *J. Pineal. Res.* 65 e12524. 10.1111/jpi.12524 30230594

[B61] Yin X. Peng J. Zhao L. Yu Y. Zhang X. Liu P. (2013). Structural changes of gut microbiota in a rat non-alcoholic fatty liver disease model treated with a Chinese herbal formula. *Syst. Appl. Microbiol.* 36 188–196. 10.1016/j.syapm.2012.12.009 23453736

[B62] Zeng X. Su W. Zheng Y. He Y. He Y. Rao H. (2019). Pharmacokinetics, tissue distribution, metabolism, and excretion of naringin in aged rats. *Front. Pharmacol.* 10:34. 10.3389/fphar.2019.00034 30761003 PMC6362423

[B63] Zeng X. Yao H. Zheng Y. Chen T. Peng W. Wu H. (2020). Metabolite profiling of naringin in rat urine and feces using stable isotope-labeling-based liquid chromatography-mass spectrometry. *J. Agric. Food Chem.* 68 409–417. 10.1021/acs.jafc.9b06494 31833363

[B64] Zhang H. J. He J. Pan L. L. Ma Z. M. Han C. K. Chen C. S. (2016). Effects of moderate and vigorous exercise on nonalcoholic fatty liver disease: a randomized clinical trial. *JAMA Intern. Med.* 176 1074–1082. 10.1001/jamainternmed.2016.3202 27379904

[B65] Zhang X. Zhan Y. Lin W. Zhao F. Guo C. Chen Y. (2020). Smurf1 aggravates non-alcoholic fatty liver disease by stabilizing SREBP-1c in an E3 activity-independent manner. *FASEB J.* 34 7631–7643. 10.1096/fj.201902952RR 32301540

[B66] Zhao L. Zhang Q. Ma W. Tian F. Shen H. Zhou M. (2017). A combination of quercetin and resveratrol reduces obesity in high-fat diet-fed rats by modulation of gut microbiota. *Food Funct.* 8 4644–4656. 10.1039/c7fo01383c 29152632

[B67] Zhou B. Liu C. Xu L. Yuan Y. Zhao J. Zhao W. (2020). N(6) - methyladenosine reader protein Ythdc2 suppresses liver steatosis via regulation of mRNA stability of lipogenic genes. *Hepatology* 10.1002/hep.31220 Online ahead of print 32150756

[B68] Zhou C. Lai Y. Huang P. Xie L. Lin H. Zhou Z. (2019). Naringin attenuates alcoholic liver injury by reducing lipid accumulation and oxidative stress. *Life Sci.* 216 305–312. 10.1016/j.lfs.2018.07.031 30031061

[B69] Zhou L. Wang Y. Xie Z. Zhang Y. Malhi S. S. Guo Z. (2018). Effects of lily/maize intercropping on rhizosphere microbial community and yield of *Lilium davidii* var. unicolor. *J. Basic Microbiol.* 58 892–901. 10.1002/jobm.201800163 30101457

