# Supplementary figures and images for: Naringin Attenuates High Fat Diet Induced Non-alcoholic Fatty Liver Disease and Gut Bacterial Dysbiosis in Mice
Source: Front Microbiol. 2020 Nov 13;11:585066. doi: 10.3389/fmicb.2020.585066 (PMC7691324; doi:10.3389/fmicb.2020.585066)

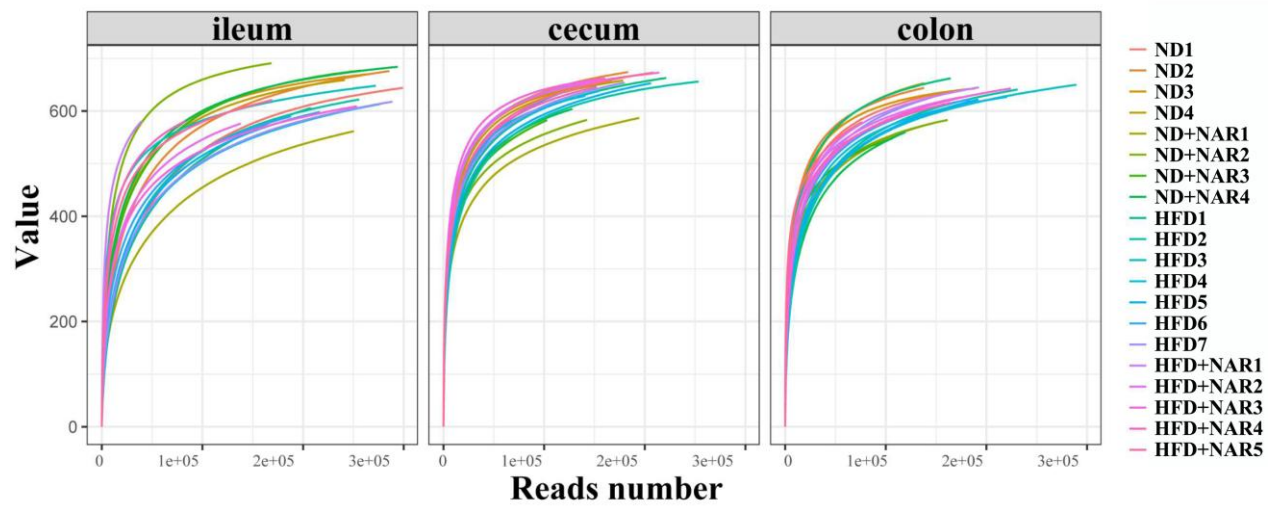

**Supplementary Fig. 1.** The rarefaction curve of all samples from ileum, cecum, colon.

Supplement: Supplementary file 1 [file Image_1.pdf]

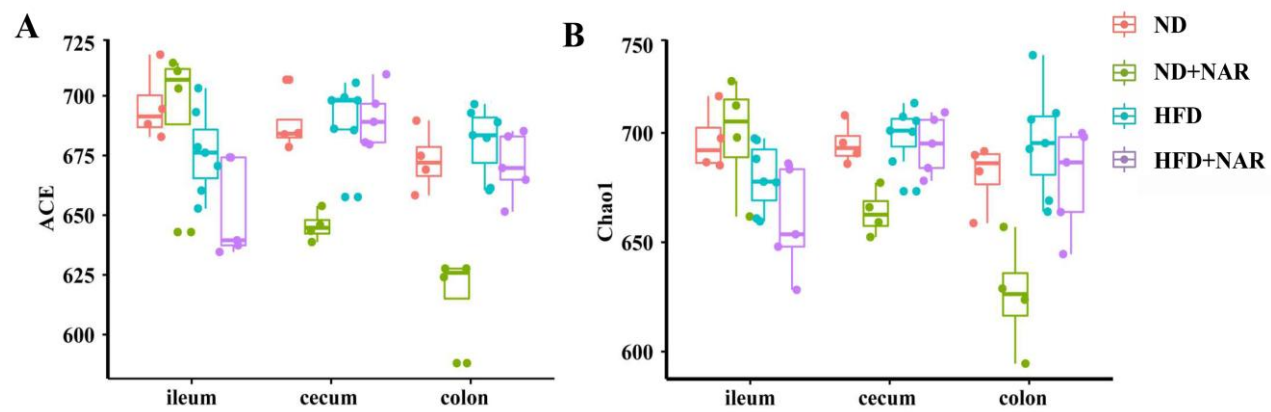

**Supplementary Fig. 2.** Alpha diversity of the gut microbiota assessed by ACE (A) and Chao1 (B).

Supplement: Supplementary file 2 [file Image_2.pdf]
